# Bee Pollen and Probiotics May Alter Brain Neuropeptide Levels in a Rodent Model of Autism Spectrum Disorders

**DOI:** 10.3390/metabo12060562

**Published:** 2022-06-18

**Authors:** Mashael A. Alghamdi, Laila Al-Ayadhi, Wail M. Hassan, Ramesa Shafi Bhat, Mona A. Alonazi, Afaf El-Ansary

**Affiliations:** 1 Department of Chemistry, Imam Mohammad Ibn Saud Islamic University (IMSIU), P.O. Box. 90950, Riyadh 11623, Saudi Arabia; mabalghamdi@imamu.edu.sa; 2 Department of Physiology, Faculty of Medicine, King Saud University, P.O. Box 2925, Riyadh 11461, Saudi Arabia; ayadh2@gmail.com; 3 Department of Biomedical Sciences, University of Missouri—Kansas City School of Medicine, 2411 Holmes Street M3-417, Kansas City, MO 64108, USA; hassanwm@umkc.edu; 4 Biochemistry Department, College of Sciences, King Saud University, P.O. Box 22452, Riyadh 11495, Saudi Arabia; rbhat@ksu.edu.sa (R.S.B.); moalonazi@ksu.edu.sa (M.A.A.); 5 Central Research Laboratory, Female Center for Medical Studies and Scientific Section, King Saud University, P.O. Box 22452, Riyadh 11495, Saudi Arabia

**Keywords:** autism spectrum disorders (ASD), neuropeptides, propionic acid, bee pollen, probiotics, fecal transplant

## Abstract

Neuropeptides play a major role in maintaining normal brain development in children. Dysfunction of some specific neuropeptides can lead to autism spectrum disorders (ASD) in terms of social interaction and repetitive behavior, but the exact underlying etiological mechanisms are still not clear. In this study, we used an animal model of autism to investigate the role of bee pollen and probiotic in maintaining neuropeptide levels in the brain. We measured the Alpha-melanocyte-stimulating hormone (α-MSH), Beta-endorphin (β-End), neurotensin (NT), and substance P (SP) in brain homogenates of six studied groups of rats. Group I served as control, given only PBS for 30 days; Group II as an autistic model treated with 250 mg PPA/kg BW/day for 3 days after being given PBS for 27 days. Groups III-VI were denoted as intervention groups. G-III was treated with bee pollen (BP) 250 mg/kg body weight/day; G-IV with *Lactobacillus paracaseii* (LB) (109 CFU/mL) suspended in PBS; G-V with 0.2 g/kg body weight/day Protexin^®^, a mixture of probiotics (MPB); and G-VI was transplanted with stool from normal animals (FT) for 27 days prior to the induction of PPA neurotoxicity on the last 3 days of study (days 28–30). The obtained data were analyzed through the use of principal component analysis (PCA), discriminant analysis (DA), hierarchical clustering, and receiver operating characteristic (ROC) curves as excellent statistical tools in the field of biomarkers. The obtained data revealed that brain levels of the four measured neuropeptides were significantly reduced in PPA-treated animals compared to healthy control animals. Moreover, the findings demonstrate the ameliorative effects of bee pollen as a prebiotic and of the pure or mixed probiotics. This study proves the protective effects of pre and probiotics against the neurotoxic effects of PPA presented as impaired levels of α-MSH, β-End, NT, and SP.

## 1. Introduction

The gut and brain are connected through numerous metabolic and signaling pathways, each with a probable impact on brain development and cognitive health [1]. Numerous studies recognize the dynamic and bidirectional interaction between the gut microbiota and their host brain via the microbiota–gut–brain axis [1]. It is well accepted that the interaction between the gut bacteria and the brain controls the development and function of the brain and is involved in neurodevelopmental disorders, such as autism spectrum disorder (ASD) [2]. There are continuous trials to find prospects to adjust and improve the microbiota as a promising strategy to enhance human health and well-being [1]. Supplementation with pro-, pre-, and phytochemicals, which may act as prebiotics, are among the most recommended, non-invasive, and safe opportunities to improve the quality of gut microbiota. Fecal microbiota transplants are another possible therapeutic strategy that could promote the colonization of donor microbiota and shift the bacterial diversity of children with ASD near that of healthy controls [3]. These strategies lead to remarkable long-term modifications of the gut microbiota in healthy volunteers, with the shift toward healthy microbiota composition, and denote a rather safe procedure for the recipients without long-term adverse events [4].

Neuropeptides, as biologically active peptides, play several roles in the bidirectional gut–brain axis pathway; these peptides could be targeted to treat certain neurological and/or gastrointestinal (GI) disorders [5]. Neuropeptides could also help understand the complex interactions between the gut and the brain [6]. Because their exact role in the microbiota–gut–brain axis has not yet been clarified, neuropeptides, such as alpha-melanocyte-stimulating hormone (α-MSH), beta-endorphin (β-End), neurotensin (NT), and substance P (SP), must be screened in different rodent models of ASD as neurodevelopmental disorders with GI co-morbidity. Sahley and Panksepp [7] and Sandman CA and Kastin AJ [8] proposed an interesting theory, which states that the altered levels of β-End, endogenous opioid peptides may alter social behavior and produce autistic-like features. Studies of brain opioid levels in autism have primarily produced inconsistent results, with plasma and CSF BE levels in autistics reported to be increased [9,10], decreased [11,12,13], or similar to controls [14]. It is interesting to know that β-End is a product of cleavage of its precursor, proopiomelanocortin (POMC)—a pre-, pro-peptide that also gives rise to Alpha-Melanocyte-stimulating hormone α-MSH as one of the chief anorexigenic neuropeptides in the brain, which acts on melanocortin (MC) type 4 receptors (MC4R) [15]. Studies in rodent models and humans have shown promising therapeutic effects of Melanotan-II (MT-II), a melanocortin receptor 4 agonist, in its ability to stimulate oxytocin production. Oxytocin can alter social cognition through its ability to modulate several neurochemical systems, including serotonin, glutamate, dopamine, and GABA neurotransmitters in the hypothalamus, amygdala, and hippocampus [16,17,18].

Neurotensin (NT) is a tridecapeptide, commonly distributed through the brain and other peripheral tissues of mammals. Altered levels of NT could be found in the brains of patients with cognitive dysfunction [19]. The antipsychotic similar effect of NT in rodent models has been somewhat attributed to the increase in brain γ-aminobutyric acid (GABA) transmission, which could help restore the imbalanced GABA/glutamate or inhibitory/excitatory imbalance, which seems to be recorded in patients with ASD [20,21]. Intra-cortical perfusion with NT changes the extracellular glutamate levels in a bell-shaped and concentration-dependent manner, indicating that NT plays a relevant role in the regulation of cortical glutamate neurotransmission [22]. Therefore, there have been conflicting reports regarding the effects of NTS, such as inhibition of GABAergic synaptic transmission or enhancement of GABAergic release in the prefrontal cortex and enhancement of GABAergic activity in the rat hippocampus [23,24,25,26]. NT may act on the central nervous system (CNS) as an atypical neuroleptic [27]. Thus, an intervention targeting NT adjustment would be a possible new therapeutic strategy to induce favorable effects on the brain in the presence of pathological conditions [27,28]. Significantly lower serum level of NT in patients with neurological disorders, such as ASD and schizophrenia, was reported [29].

Substance P was among the reported neuropeptides that exert neuroprotective effects on the brain, mainly through preventing Aβ accumulation, increasing neuronal glucose transport, increasing the production of neurotrophins, inhibiting endoplasmic reticulum stress and autophagy, modulating potassium channel activity, and hippocampal long-term potentiation; therefore, neuropeptides may function as potential drug targets in the prevention and cure of neurological disorders, such as Alzheimer’s disease [30]. Moreover, Stumm et al. [31] suggested that the promotion of overexpression of SP in GABAergic neurons enhances GABAergic inhibitory circuits, which may result in endogenous neuroprotection against hyperexcitation.

Malfunctioning or leaky blood–brain barrier (BBB) is a well-accepted phenomenon in ASD. It is evidenced by the presence of circulating autoantibodies directed against the fetal brain proteins and neuropeptides [32,33].

Based on this information, bee pollen as a prebiotic, Lactobacillus as a probiotic, and fecal transplant as three protective strategies could improve brain function of ASD patients indirectly through gut. The amendment of gut permeability and microbial dysbiosis, as two autistic features related to the gut, could be associated with the amelioration of neuroinflammation, glutamate excitotoxicity, and oxidative stress as the three major etiological mechanisms in ASD. This could partially rescue the impaired social behavior as the main clinical presentation of ASD [4]. Use of translational research to understand the role of α-MSH, β-End, NT, and SP in relation to different etiological mechanisms of ASD could help identify new preventive or therapeutic targets for the management of this disorder [1,2,3,4].

This information initiates our interest to measure the brain levels of β-End, α-MSH, NT, and substance P in brain homogenates of PPA-induced rodent model of ASD and test the potency of bee pollen, probiotics, and fecal transplant in ameliorating the neurotoxic effects of PPA through the use of principal component analysis (PCA), discriminant analysis (DA), hierarchical clustering, and ROC curves as excellent statistical tools in the field of biomarkers.

## 2. Results and Discussion

**Figure 1:**
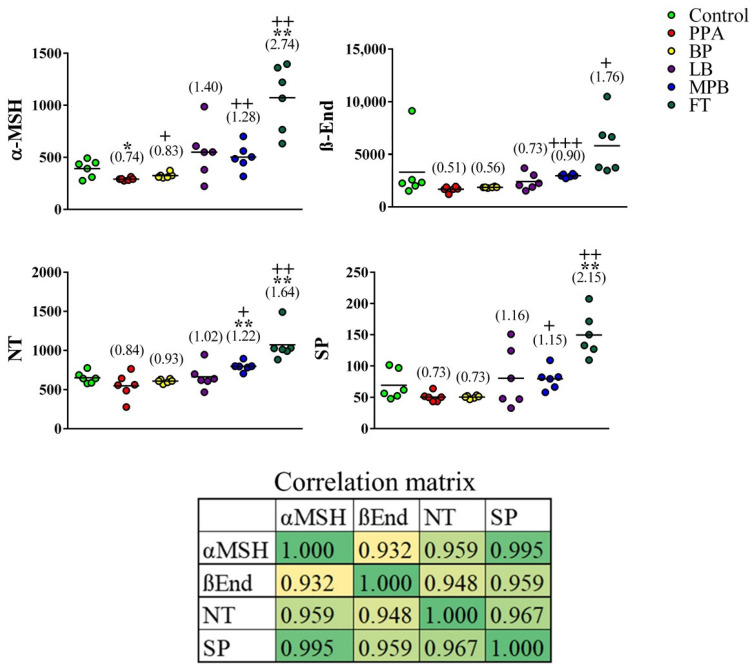
Effects of bee pollen and probiotic intestinal microbiota on neuropeptide levels in a rat model of autism. Unpaired *t*-test was used to test the significance of differences between the control group and each of the remaining groups. Corresponding *p* values of <0.05 and <0.005 are indicated by “*” and “**”, respectively. Differences between the PPA group and each of the other non-control groups were similarly tested, and corresponding *p* values of <0.05, <0.005, and <0.0005 are indicated by “+”, “++”, and “+++”, respectively. Fold change relative to controls is indicated in parentheses. PPA: propionic acid; BP: bee pollen; LB: *Lactobacillus*; MPB: mixed probiotic bacteria; FT: fecal transplant. The correlation matrix shows correlations between the neuropeptide levels (bottom). Correlation was calculated using Pearson product–moment correlation analysis. The heatmap shows r values. The *p* values associated with r at 95% confidence interval are 0.007, 0.002, 0.00004, 0.004, 0.00249, and 0.00158 for α-MSH/β-End, α-MSH/NT, α-MSH/SP, β-End/NT, β-End/SP, and NT/SP, respectively.

α-MSH levels were significantly lower in PPA-treated animals than in controls. This effect was reversed by bee pollen and mixed probiotic bacteria (α-MSH was significantly higher in both groups than in the PPA group and was not significantly different from the control group) and overturned by fecal transplants (α-MSH was significantly higher in the transplant group than in both the control and PPA groups) (Figure 1). β-End, NT, and SP levels showed lower means in PPA-treated rats than in controls; however, the differences were not statistically significant. In addition to dramatically boosting α-MSH levels (2.7-fold), fecal transplants appeared to enhance brain levels of NT and SP to a level that was above that of controls (1.6-fold and 2.2-fold, respectively). NT levels were 1.2-fold higher in the probiotic group than in the control group (Figure 1). It is clear that the variance in data in the FT-treated group is large, presented as more scattered distribution around the mean, while the variance in data in the BP, LP, and MPB is small, so the data set is clustered. This could be attributed to special effects of the non-bacterial fecal components and functional interactions between bacterial populations [3,4].

The neurotoxic effect of PPA in the present study represents a remarkable decrease in the four measured neuropeptides. The validity of our rodent model in relation to the remarkable decrease in α-MSH can be supported by the work of Dang et al. [34] in which they reported that social isolation for a period of 6 weeks caused a drastic reduction in α-MSH-immunoreactivity in different brain areas known to be generally involved in the pathogenesis of social isolation [35]. Re-socialization of the socially isolated rats, over a period of 72 h, led to a full recovery of the α-MSH-immunoreactivity profile, concomitant with complete attenuation of the anxiety- and depression-like behaviors [36].

**Figure 2:**
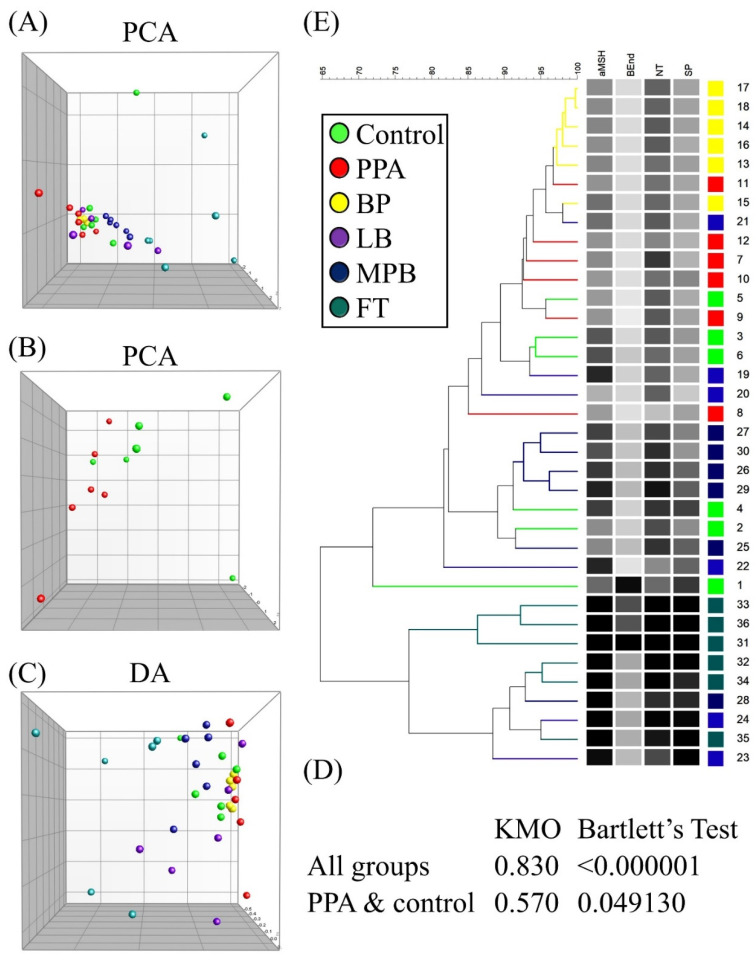
Clustering of treatment groups using principal component analysis (PCA) (**A**,**B**), discriminant analysis (DA) (**C**), and hierarchical clustering (**E**). PC1 and PC2 in PCA and DA are represented by the *x* and *y* axes, respectively. Kaiser–Meyer–Olkin (KMO) and Bartlett’s test of sphericity results are shown for all groups and PPA and control groups (**D**).

Although α-MSH was the only neuropeptide that was significantly different in the PPA group compared to the control group, each of the other neuropeptides (Figure 1) showed a downward trend that mimics α-MSH trajectory in PPA-treated animals. For this reason, we decided to test whether any of the other neuropeptides together with α-MSH might improve the latter’s ability to predict autism-like disease in our animal model. We first performed a PCA on all groups, which showed that group separation was mainly spread out over PC1 axis (Figure 2A). As expected, variable contributions to the most discriminating PC (i.e., PC1) showed that α-MSH contributed the most to PC1, but other neuropeptides, especially NT and SP, also contributed substantially (Table 1).

PCA of the PPA and control groups showed substantial group separation on both PC1 and PC2 axes (Figure 2B). PCA on all groups passed both KMO and Bartlett’s tests, while that on the PPA/control group failed both tests (Figure 2D). Monte Carlo simulation results suggested that PC1 in each case was the only significant PC (Figure S1). The DA of all groups showed similar results as those of PCA (Figure 2C and Table 2). DA of the PPA and control groups resulted in one PC accounting for all variance (no plot was generated, since we only had one axis), with α-MSH being the top contributor to this PC (Table 2).

**Figure 3:**
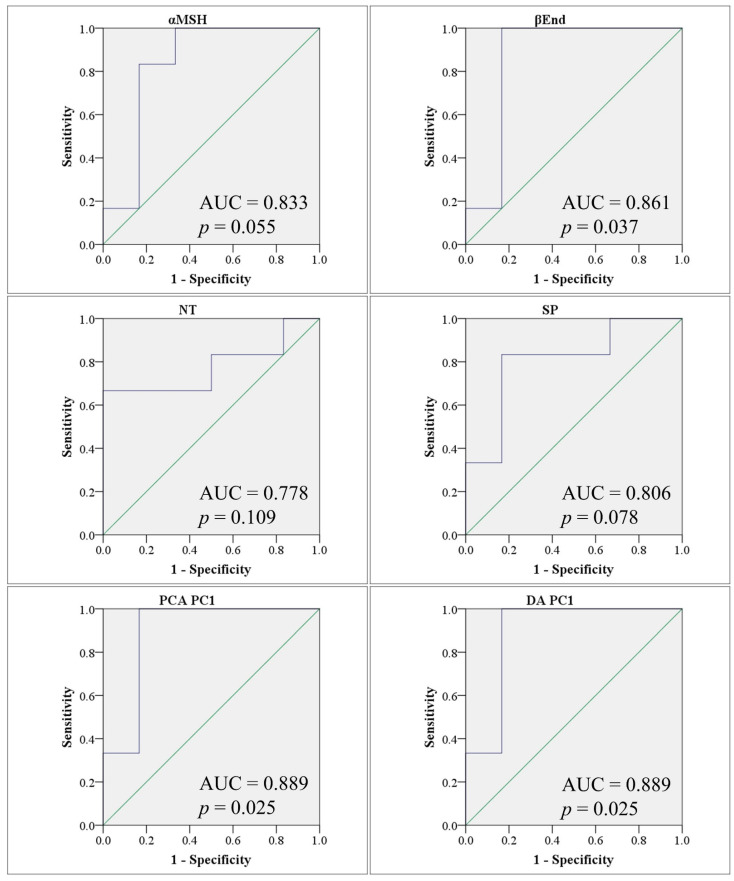
Receiver operating characteristic (ROC) curves showing area under the curve (AUC) obtained using individual biomarkers (top two rows), principal component analysis PC1 scores (PCA PC1) (bottom left), and discriminant analysis PC1 scores (DA PC1) (bottom right) to differentiate between PPA and control animals. AUC values and significance p values are shown for each ROC curve. ROC curves are shown in blue and the diagonals (marking an AUC of 0.5) are shown in green.

Hierarchical clustering results were consistent with those of PCA and DA, that is, they showed that BP was the closest to PPA, while MPB and FT were the farthest. All three tests agreed that noticeable group separation exists with significant overlap (Figure 2E). Our results so far were consistent with the possibility that β-End, NT, and SP may have contributed enough variance to allow the use of these neuropeptides as potential markers of the autism-like disease in our animal model (Table 3 and Figure 3).

The significant decrease in β-End level observed in the present study can demonstrate the neurotoxic effect of PPA. It is well known that β-End plays central roles in enthusiasm, sensation, normal social interaction, response to stress, intellectual function, and pain [37,38]. Fujii et al. [39] hypothesized that β-End deficiencies might be involved in multiple neurological disorders and that neuropeptides can be linked to brain neurotransmitters. This can support the neuroprotective effects of BP, LP, MPB, and FT, the four tested intervention strategies in the present study, and their considerable effectiveness in ameliorating the PPA-induced depletion of β-End.

The remarkable decrease in NT in PPA-treated rats is in good agreement with the study performed by Nelson et al. [40], which demonstrates significantly lower NT in subjects with ASD than in controls. The ameliorative effects of the four used intervention strategies led to a remarkable increase in NT; this result can be supported by the fact that NT, as a commonly circulated neuromodulator in the brain and peripherally, has important roles in cognition. Additionally, administration of NTS1-receptor agonist has beneficial actions in rat and mouse models of neurological disorders [41,42]. The protective effects of microbiota-related intervention strategies (LP, MPB, and FT) can find support in a recent report by Fetissov et al. [43], which demonstrates the involvement of neuropeptides in the regulation of feeding and social behaviors through the gut-microbiota–brain axis. This might suggest the usefulness of LB, MPB, and FT as intervention strategies to correct the GI co-morbidities, abnormal feeding behavior, and social interaction impairment associated with ASD. Contrary to the inflammatory effect of NT, recently, Tsilioni et al. [44] recorded a key finding that NT increases the gene expression of the anti-inflammatory IL-37 in cultured human microglia. This might support the protective effects of BP, LB, MPB, and FT recorded in the present study (Figure 1).

Mostafa et al. [45] reported significantly higher levels of neurokinin A receptor, the specific receptor of SP, in the serum of children with ASD; they also reported that this level is significantly correlated with the severity of autism. This result indirectly supports the PPA-induced decrease in brain SP. It is well known that individuals with ASD have disrupted BBB, and hence, lower serum SP level could be concomitant with much lower brain neuropeptides levels [32,33].

The effects of β-END on brain and behavior can be understood on the basis that β-END, which is released into the CSF, can affect different distant brain areas that are involved in a variety of behaviors related to reward processing and motivational and mental conditions. As a global effect, this usually helps reduce stress, leading to a sense of well-being by homeostatic balance and behavioral stability.

It is well known that alterations of gut microbiota are associated with increased gut permeability, or “leaky gut”, which permits bacterial metabolites to cross the gut barrier, inducing abnormal brain neurodevelopment during early childhood in vulnerable children through the gut–brain axis [3,4]. This might support the remarkable protective effects of the four studied intervention strategies of the present study, as they are directly related to gut homeostasis. This can find support through considering the work of Tungland [46], which proves that utilization of pure or mixed probiotics and prebiotics and transplantation of fecal microbiota have shown significant benefits in preventing and reversing the illnesses related to brain-to-gut and gut-to-brain malfunction along the bidirectional gut–brain axis.

### Limitations

The value of combining biomarkers is not clear from the current data. At 100% sensitivity, the specificity is lower than independent α-MSH. However, the sample size is small in the current study, which means a bigger sample is needed to determine whether other neuropeptides are valuable and whether a multivariate combination biomarker is better than α-MSH alone.

KMO was less than 0.7, and Bartlett’s test *p* value was greater than 0.001 for the PPA/control group. We tried resolving this issue by combining the control group with either LB alone or LB + BP. KMO results remained below 0.7 and Bartlett’s *p* value above 0.001 (data not shown).

## 3. Materials and Methods

### 3.1. Materials

#### Prebiotic, Probiotic, and Fecal Transplant

Bee pollen was purchased from a branch of Wadi Al-Nahil in Riyadh, Saudi Arabia, in June 2019, under the trade name “bee pollen, 100% natural bee pollen first elite”. Wadi Al-Nahil, one of the largest marketing companies in Saudi Arabia (www.wadialnahil.net) accessed on 10 March 2021, imported it. The major compounds in the bee pollen sample used in the current study include polyphenols, mainly flavonoids, and proanthocyanidins, ethyl ester of hexadecanoic acid, eicosatrienoic acid, 1,4-dimethyl-benzene, hexadecanoic acid (palmitic acid), and nonacosane according to the previously published analysis by Al-Yousef et al. [47].

*Lactobacillus paracaseii* (Strain LPC-37), a product of Life Extension, was purchased. Powder from 1 capsule, which contains 5 × 10^9^ colony forming units (CFU) per 25 mg, was dissolved in 1 mL sterile PBS. Animals were given 0.2 mL daily (1 × 10^9^ CFU) by oral gavage.

ProtexinR (Somerset, UK) is a mixture of some healthy bacteria, including *Bifidobacterium infantis*, *Bifidobacterium breve*, *Lactobacillus acidophilus*, *Lactobacillus bulgaricus*, *Lactobacillus casei*, *Lactobacillus rhamnosus*, *Streptococcus thermophiles*, with the concentration of 1 billion CFU per gram.

For the fecal transplant, 1 g of pooled fecal samples from healthy donor rats was suspended in 10 mL of sterile PBS, pH 7.4, through vortexing. The homogenized solution was then filtered twice using a sterilized metal sieve. Fecal transplantation was performed by rectal infusion of the fecal filtrate at a dose of 1 g/kg [48].

### 3.2. Animals

The present study’s experiments were carried out on 36 three-week-old male Wister albino rats weighing 60–80 grams. The experimental procedure was pre-approved by the ethics committee for animal research of King Saud University, Riyadh (ethics reference number: KSU-SE-19-35). Rats were randomly divided into six groups, six rats in each group. All the rats were individually housed in cage 41 (40 × 35 × 20 cm^3^) at a temperature of 21 ± 1 °C and light–dark cycle of 12:12 h (light on at 9:00, light off at 21:00). Animals had free access to food (standard laboratory animal feed pellets) and water.

### 3.3. Study Design

Pre-determination of sample size was not performed. Enrolled animals were randomly allocated to 6 groups (6 animals/group). The study was designed to be performed over 30 days. The animals were administered PBS for 30 days (control group); treated with PBS for the first 27 days followed by that with 250 mg PPA/kg BW/day for 3 days (PPA group; autistic model); or administered bee pollen (BP) 250 mg/kg body weight/day (bee pollen group), administered *Lactobacillus paracaseii* (LB)(10^9^ CFU/mL) suspended in PBS (phosphate-buffered solution pH 7.2) (LP group), administered 0.2 g/kg body weight/day Protexin^®^ (a mixture of probiotics (probiotic group) (MPB); or transplanted with stool from normal animals (FT group) for 27 days prior to the induction of PPA neurotoxicity on the last 3 days. The transplants were performed anorectally after suspending the stool samples in PBS solution at a pH of 7.2. Graphical schemes illustrating the animal groups and treatments are presented as Figure S2.

### 3.4. Preparation of Brain Homogenate for the Identification of Neuropeptides

At the end of the feeding periods, the rats were anesthetized with carbon dioxide and euthanized. All the animals were killed after 30 days of study; the whole brain tissue was removed, washed with distilled water, and homogenized in phosphate buffer 1:10 *w*/*v* using Tissue Lyser LT (QIAGEN) with high-speed shaking in micro centrifuge tubes.

### 3.5. Quantification of the Neuropeptides in Brain Tissue

Concentrations of α-MSH, β-End, NT, and SP were measured in the brain tissue homogenate by using MILLIPLEX^®^ MAP kit for rat neuropeptides Magnetic Bead panel, according to the manufacturer’s instructions.

### 3.6. Statistical Analysis

Significance of differences between groups was tested using unpaired *t*-tests. Correlations between variables were calculated using Pearson’s product–moment correlation. Both tests were performed using GraphPad Prism version 6.07 (GraphPad Software, La Jolla, CA, USA).

#### 3.6.1. Principal Component Analysis and Discriminant Analysis

Principal component analysis (PCA) is a statistical technique that simplifies graphical presentation of data to facilitate the display and interpretation of multivariate results. PCA calculates orthogonal (i.e., perpendicular) eigenvectors, which can also be called principal components (PCs), and scores. The first eigenvector is chosen, so that it explains the most variance, while the second is an orthogonal eigenvector that explains the largest portion of the remaining variance. Additional eigenvectors are sequentially computed in a descending order of the amount of variance they explain and on the condition of orthogonality. The top two or three PCs are rotated, so that they form a new 2D or 3D coordinate system—composed of *x* and *y* axes or *x*, *y*, and *z* axes, respectively—within which data points are plotted using the scores calculated for each of them. There can be as many PCs as original variables, but only the top ones that account for most of the variance are included in the study. Additionally, Monte Carlo simulation is used to identify statistically significant PCs, which are the only ones used in data interpretation. In the current study, two transformations were applied before performing PCA. Subtraction of average over variables was applied to central data points around the origin, and division by variance over variables was used to equalize the power of variables. The latter transformation (i.e., scaling to variance) is particularly important for dealing with variables that show widely different means to avoid over emphasizing the effect of variables whose means are relatively large at the expense of other variables with smaller means [49]. We used the Kaiser–Meyer–Olkin (KMO) measure of sampling adequacy to evaluate our sample size with a cutoff value of 0.7 [50,51]. Bartlett’s test of sphericity was used to test the probability that our variables are orthogonal, which means that the covariance matrix is an identity matrix (i.e., a covariance matrix with all ones in the diagonal and all zeros elsewhere). PCA is informative only in the presence of some correlations between variables; therefore, the null hypothesis stating that such correlations are absent is rejected in Bartlett’s test at a significance threshold of *p* ≤ 0.001 [52]. We also used discriminant analysis (DA) to identify the most important variables in distinguishing between PPA and control animals. DA computes principal components as well. A major difference between PCA and DA is that PCA designs its PCs to account for the most variance, without any consideration of predefined group memberships. DA defines its PCs in such a way that it maximizes separation between groups [49], which makes DA more powerful in identifying the discriminating power of various variables by defining their contributions to PCs. One of the assumptions of DA is the equivalence of group covariance matrices, which we tested using Box’s M test with a *p* value cutoff of 0.001 (i.e., *p* > 0.001 suggests equal matrices). PCA and DA were performed using BioNumerics version 6.6 (Applied Maths, Austin, TX, USA) or SPSS version 24.0 (IBM SPSS Statistics for Windows, Armonk, NY, USA: IBM Corp.). KMO, Bartlett’s test of sphericity, Monte Carlo simulation (Brian O’Connor’s syntax [53]), and Box’s M test were performed using SPSS.

#### 3.6.2. Hierarchical Clustering

Hierarchical clustering arranges data points in the form of a tree, so that the most similar data points are brought together on common or close branches, while distant ones are separated on different branches. In the current study, we used the Canberra metric (Equation (1)) to compute multivariate similarity; trees were constructed using the unweighted pair group method with arithmetic mean (UPGMA) algorism. Hierarchical clustering was performed using BioNumerics.

(1) $$D=\frac{1}{n}\;\sum_{i=1}^{n}\frac{\left|Xi-Yi\right|}{\left|Xi+Yi\right|}$$

where “*D*” is the Canberra metric, “*n*” is the number of variables, “*i*” is the *i*th variable, and “*X*” and “*Y*” are subjects.

#### 3.6.3. Receiver Operating Characteristic Curves

We used ROC curves to evaluate the predictive power of biomarkers. An ROC curve is generated for a given biomarker by plotting false positive (1-specificity) and true positive (sensitivity) rates associated with the range of biomarker values on the *x* and *y* axes, respectively. A perfect biomarker with 100% sensitivity and 100% specificity (i.e., 1-specificity is equal to zero for all biomarker values) will have an area under the curve (AUC) of 1.0, while a biomarker with an AUC of 0.5 is considered useless [54]. ROC curves demonstrate the tradeoff between sensitivity and specificity at various biomarker cutoff values. ROC curves were generated using SPSS.

## 4. Conclusions

This work highlighted the importance of neuropeptides as biomarkers of dysregulated gut–brain axis and altered gut microbial diversity as an etiological mechanism of autism. Moreover, it highlighted the possibility of using prebiotics (BP), pure *Lactobacillus paracaseii* (LP), mixed probiotics (MP), and fecal transplant (FT) as protective intervention strategies to avoid the neurotoxic effect of PPA, an SCFA acid related to the pathoetiology of autism.

## Figures and Tables

**Table 1:** Contribution of variables to PC1 and PC2 in principal component analysis. Note: while determining variable contributions to principal components, only the magnitude of the contribution is considered, with no regard for directionality (i.e., plus or minus sign) because contributions in either the positive or negative direction equally explain the variance.

All Groups				PPA and Control			
PC1 (80.81%)		PC2 (11.15%)		PC1 (55.23%)		PC2 (27.83%)	
α-MSH	5.697	β-End	3.414	SP	3.168	NT	2.802
SP	5.650	NT	−1.940	α-MSH	2.742	β-End	−1.960
NT	5.307	SP	−0.619	β-End	2.569	α-MSH	1.083
β-End	4.879	α-MSH	−0.504	NT	1.535	SP	−0.706

**Table 2:** Contribution of variables to PC1 and PC2 in discriminant analysis.

All Groups				PPA and Control	
PC1 (97.54%)		PC2 (1.52%)		PC1 (100%)	
α-MSH	−2.784	NT	0.374	α-MSH	−1.592
NT	−2.422	α-MSH	−0.340	SP	−1.032
SP	−2.326	β-End	0.239	NT	−0.782
β-End	−1.515	SP	−0.138	β-End	−0.751

**Table 3:** Evaluation of the utility of four neuropeptides in predicting an autism-like disease in a PPA model of ASD using ROC analysis (PPA: n = 6, control: n = 6). PCA: first principal component in principal component analysis; DA: first principal component in discriminant analysis.

ROC Analysis	AUC	*p* Value	Cutoff	Sensitivity (%)	Specificity (%)
PCA	0.889	0.025	−1.45	100	83.3
DA	0.889	0.025	0.35	100	83.3
α-MSH	0.833	0.055	301	83.3	83.3
β-End	0.861	0.037	1965	100	83.3
NT	0.778	0.109	645	83.3	50.0
SP	0.806	0.078	52	83.3	83.3

## Data Availability

The data presented in this study are available in article and supplementary material.

## Supplementary Materials

The following supporting information can be downloaded at: https://www.mdpi.com/article/10.3390/metabo12060562/s1, Figure S1: Evaluation of principal component significance using Monte Carlo simulation. Principal compo-nents and their corresponding eigenvalues are plotted for observed data (blue lines), 50th percen-tile (green lines), and 95th percentile (red lines) simulated data. Significant principal components in the observed data must have higher eigenvalues than the corresponding simulated principal components, Figure S2: Graphical scheme illustrating the animal groups as control (G1), PPA-rodent model (G2), BP, LP, MPB, and FT pre-protected PPA-rodent model (Gs 3-6).

## Abbreviations

Autism spectrum disorders (ASD); Alpha-melanocyte-stimulating hormone (α-MSH); Beta-endorphin (β-End); bee pollen (BP); blood–brain barrier (BBB); central nervous system (CNS); discriminant analysis (DA); fecal transplant (FT); γ-aminobutyric acid (GABA); melanocortin (MC); Melanotan-II (MT-II); mixture of probiotics (MPB); neurotensin (NT); phosphate-buffered saline (PBS); principal component analysis (PCA); Propionic acid (PPA); proopiomelanocortin (POMC); receiver operating characteristic (ROC); substance P (SP); Short-chain fatty acids (SCFA); Statistical Package for the Social Sciences (SPSS); unweighted pair group method with arithmetic mean (UPGMA).
