# Supplementary material for: Bee Pollen and Probiotics May Alter Brain Neuropeptide Levels in a Rodent Model of Autism Spectrum Disorders
Source: Metabolites. 2022 Jun 18;12(6):562. doi: 10.3390/metabo12060562 (PMC9230532; doi:10.3390/metabo12060562)
Supplement: Supplementary file 1 [file metabolites-12-00562-s001.zip › metabolites-1771222-supplementary.pdf]

Supplementary figures

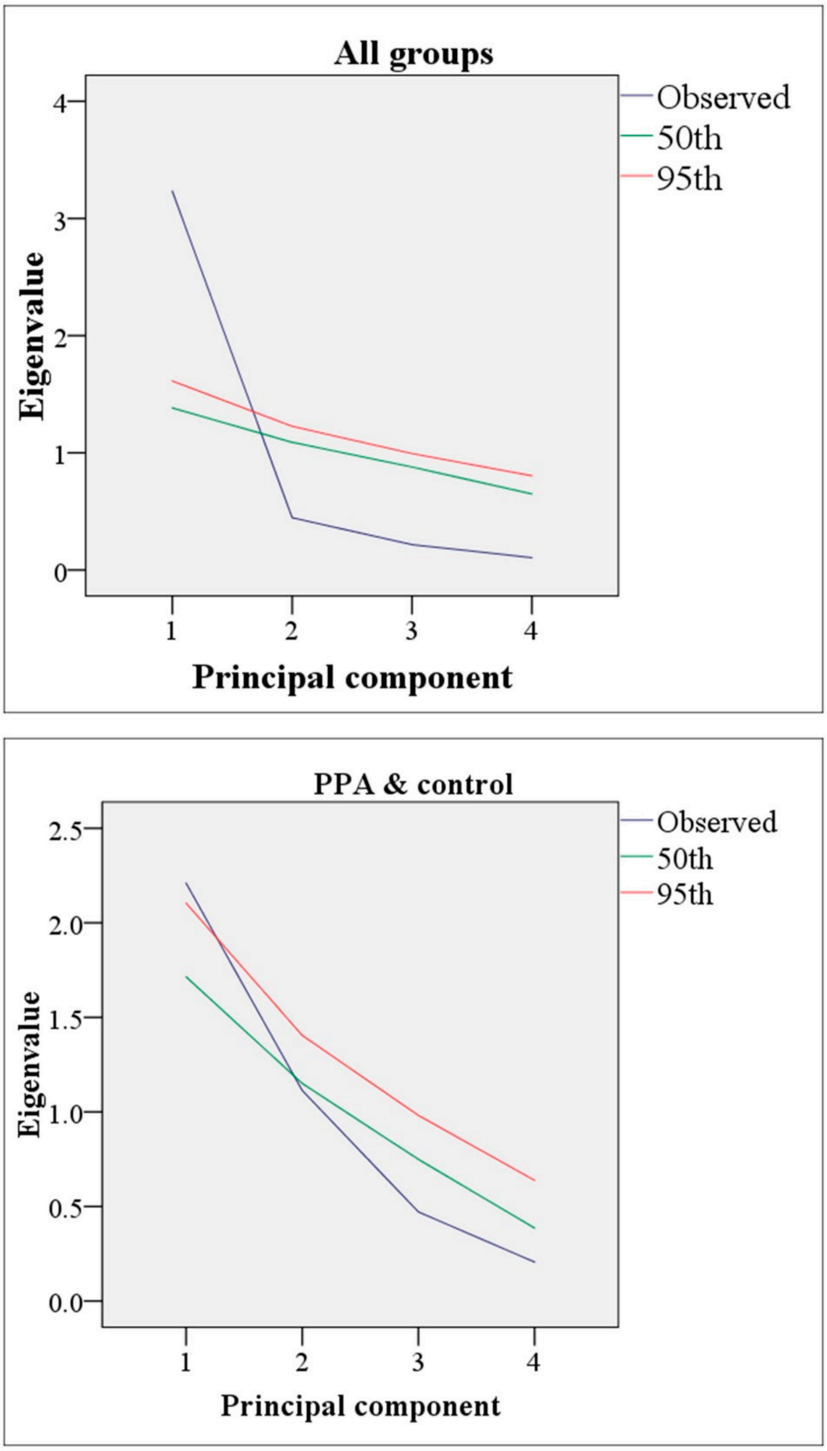

Figure S1. Evaluation of principal component significance using Monte Carlo simulation. Principal components and their corresponding eigenvalues are plotted for observed data (blue lines), 50th percentile (green lines), and 95th percentile (red lines) simulated data. Significant principal components in the observed data must have higher eigenvalues than the corresponding simulated principal components.

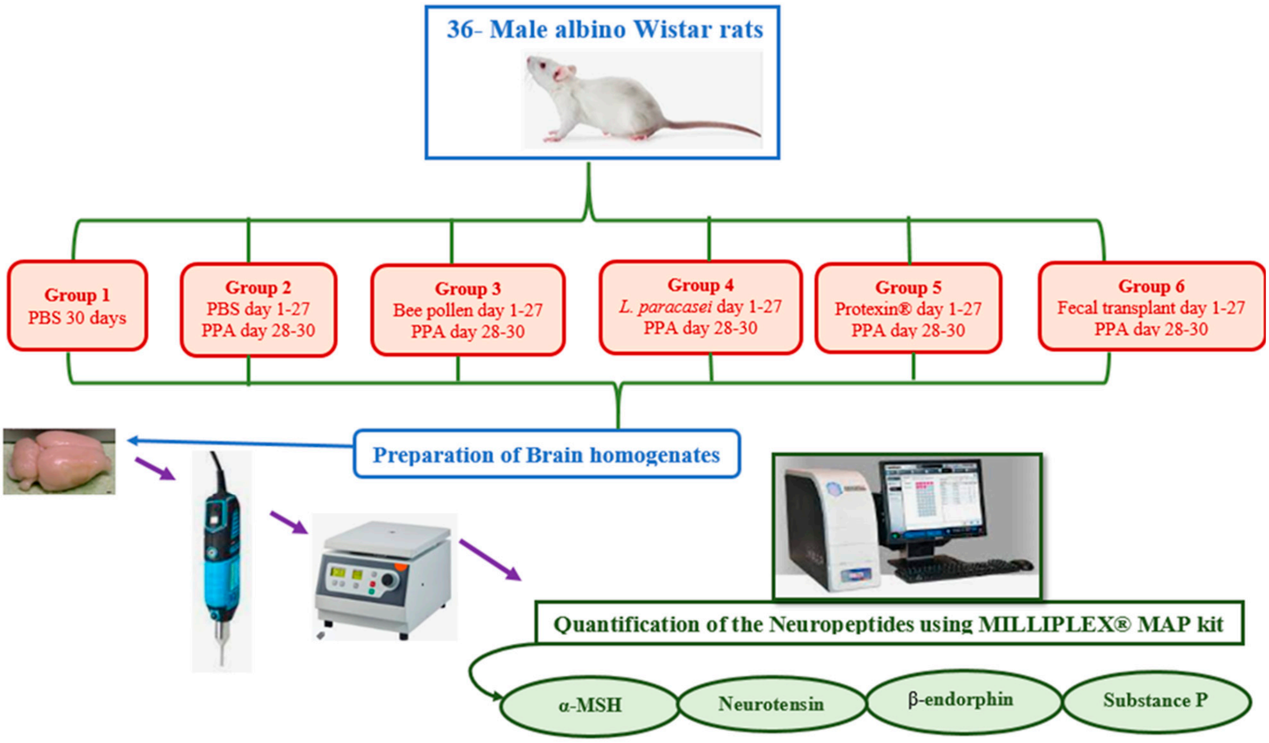

Figure S2. Graphical scheme illustrating the animal groups as control (G1), PPA-rodent model (G2), BP, LP, MPB, and FT pre-protected PPA-rodent model (Gs 3-6).
